# Supplementary material for: EnzML: multi-label prediction of enzyme classes using InterPro signatures
Source: BMC Bioinformatics. 2012 Apr 25;13:61. doi: 10.1186/1471-2105-13-61 (PMC3483700; doi:10.1186/1471-2105-13-61)
Supplement: Addtional file 5 — The Java code to format the data files, evaluate and predict. The file enzml_java_code.tar.gz contains the Java code used to format database data to ARFF and XML formats, to execute cross and train-test (jackknife) evaluations and to record evaluation results to database. More information is included in the readme.txt file and the Javadoc files. The code can be used with a MySQL database. To use a different database software, other JDBC drivers might be required. [file 1471-2105-13-61-S5.gz › java_code/utils/doc/uk/ac/ed/inf/utils/database/SqlUtils.html]

SqlUtils


---


|  |  |  |
| --- | --- | --- |
| **Overview** \| **Package** \| **Class** \| **Use** \| **Tree** \| **Deprecated** \| **Index** \| **Help** | |  |
| **PREV CLASS**   **NEXT CLASS** | **FRAMES**    **NO FRAMES**     **All Classes** |  |
| SUMMARY: NESTED \| FIELD \| CONSTR \| METHOD | DETAIL: FIELD \| CONSTR \| METHOD |  |


---


## uk.ac.ed.inf.utils.database Class SqlUtils

```
java.lang.Object
  uk.ac.ed.inf.utils.database.SqlUtils
```

---

``` public class SqlUtils extends java.lang.Object ```

Class

**Version:**
:   29 Jul 2008

**Author:**
:   Luna De Ferrari luna.deferrari-at-ed.ac.uk

---

| **Field Summary** | |
| --- | --- |
| `static int` | `DOUBLE`             Basic data type: double (passes without error a parseDouble action) |
| `static int` | `INTEGER`             Basic data type: integer (passes without error a parseInteger action) |
| `static int` | `MAX_BIGINT_LENGTH`             Maximum length in characters for a signed SQL INT BIGINT[(M)] A large integer. |
| `static int` | `MAX_CHAR_LENGTH`             Maximum length in characters for an SQL CHAR column CHAR(M) A fixed-length string. |
| `static int` | `MAX_INT_LENGTH`             Maximum length in characters for a signed SQL INT INT[(M)] A normal-size integer. |
| `static int` | `NULL`             Basic data type: null (the type could not be guessed, eg. |
| `static int` | `STRING`             Basic data type: string (anything that is not an integer or a double...) |


**Constructor Summary**

`SqlUtils()`


| **Method Summary** | |
| --- | --- |
| `static java.lang.String` | `getSelectStarSql(java.lang.String table)`             Generates a select \* query text (eg. |
| `static java.lang.String` | `getSelectWhereEqualSql(java.lang.String table, java.lang.String column, java.lang.String equalityConditionValue)`             gets sql code for a select on one column of a table, with a where x = y condition specific for that column |
| `static java.lang.String` | `getSelectWhereEqualSql(java.lang.String table, java.lang.String column1, java.lang.String equalityConditionValue1, java.lang.String column2, java.lang.String equalityConditionValue2)`             Select on two columns of a table, with two "where" conditions, , one for each provided column |
| `static java.lang.String` | `getSelectWhereLikeSql(java.lang.String table, java.lang.String column, java.lang.String equalityConditionValue)`             Gets sql code for a select on one column of a table, with a where x like y condition specific for that column |
| `static java.lang.String` | `getSelectWhereSql(java.lang.String table, java.lang.String column, java.lang.String whereCondition)`             gets sql code for a select on one column of a table, with a where condition |
| `static java.lang.String` | `getSimpleSelectSql(java.lang.String table, java.lang.String column)`             gets sql code for a select on one column of a table |
| `static java.lang.String` | `getSqlForSelectLike(java.lang.String table, java.lang.String column, java.lang.String likeColumn, java.lang.String likeCondition)`             Sql code for a select "LIKE" |
| `static java.lang.String` | `getSqlSelectByIdByRegExp(java.lang.String table, java.lang.String valueColumn, java.lang.String idColumn, java.lang.String id, java.lang.String regExp)`             Generates the sql command for a select of a value corresponding to a certain id AND matching a certain regex string |
| `static java.lang.String` | `getSqlSelectByRegExp(java.lang.String table, java.lang.String column, java.lang.String regExp)`             Generates the sql command for a select matching a certain regex string |
| `static java.lang.String` | `getSqlToDropTable(java.lang.String tableName)`             Get sql to drop table |
| `static java.lang.String` | `surroundWith(java.lang.String text, java.lang.String surrounding)`             Adds a surrounding string (usually an apex or similar) as prefix and suffix of the given text. |

**Methods inherited from class java.lang.Object**

`equals, getClass, hashCode, notify, notifyAll, toString, wait, wait, wait`

**Field Detail**

### DOUBLE

```
public static final int DOUBLE
```

:   Basic data type: double (passes without error a parseDouble action)

    **See Also:**: Constant Field Values

---


### INTEGER

```
public static final int INTEGER
```

:   Basic data type: integer (passes without error a parseInteger action)

    **See Also:**: Constant Field Values

---


### MAX\_BIGINT\_LENGTH

```
public static final int MAX_BIGINT_LENGTH
```

:   Maximum length in characters for a signed SQL INT BIGINT[(M)] A large
    integer. The signed range is -9223372036854775808 to 9223372036854775807.
    The unsigned range is 0 to 18446744073709551615.
    http://dev.mysql.com/doc/refman/5.1/en/numeric-type-overview.html

    **See Also:**: Constant Field Values

---


### MAX\_CHAR\_LENGTH

```
public static final int MAX_CHAR_LENGTH
```

:   Maximum length in characters for an SQL CHAR column CHAR(M) A
    fixed-length string. The range of M is 0 to 255 characters.

    **See Also:**: Constant Field Values

---


### MAX\_INT\_LENGTH

```
public static final int MAX_INT_LENGTH
```

:   Maximum length in characters for a signed SQL INT INT[(M)] A normal-size
    integer. The signed range is -2147483648 to 2147483647. The unsigned
    range is 0 to 4294967295.
    http://dev.mysql.com/doc/refman/5.1/en/numeric-type-overview.html

    **See Also:**: Constant Field Values

---


### NULL

```
public static final int NULL
```

:   Basic data type: null (the type could not be guessed, eg. the list was
    empty)

    **See Also:**: Constant Field Values

---


### STRING

```
public static final int STRING
```

:   Basic data type: string (anything that is not an integer or a double...)

    **See Also:**: Constant Field Values


**Constructor Detail**

### SqlUtils

```
public SqlUtils()
```


**Method Detail**

### getSelectStarSql

```
public static java.lang.String getSelectStarSql(java.lang.String table)
```

:   Generates a select \* query text (eg. SELECT \* FROM mytable )

    :   **Parameters:**: `table` - **Returns:**: a string containing the sql query text

---


### getSelectWhereEqualSql

```
public static java.lang.String getSelectWhereEqualSql(java.lang.String table,
                                                      java.lang.String column,
                                                      java.lang.String equalityConditionValue)
```

:   gets sql code for a select on one column of a table, with a where x = y
    condition specific for that column

    :   **Parameters:**: `table` - the table to select from: `column` - the column to select from: `equalityConditionValue` - the x value for the: where column = 'x' condition. **Returns:**: sql code for a select on one column of a table, with a where condition specific for that column

---


### getSelectWhereEqualSql

```
public static java.lang.String getSelectWhereEqualSql(java.lang.String table,
                                                      java.lang.String column1,
                                                      java.lang.String equalityConditionValue1,
                                                      java.lang.String column2,
                                                      java.lang.String equalityConditionValue2)
```

:   Select on two columns of a table, with two "where" conditions, , one for
    each provided column

    :   **Parameters:**: `table` - the table to select from: `column1` - the column to select from: `equalityConditionValue1` - the x1 value for the: where column1 = 'x1' condition.: `column2` - the column to select from: `equalityConditionValue2` - the x2 value for the: where column2 = 'x2' condition. **Returns:**: SQL for a select on two columns of a table, with two "where" conditions, one for each provided column

---


### getSelectWhereLikeSql

```
public static java.lang.String getSelectWhereLikeSql(java.lang.String table,
                                                     java.lang.String column,
                                                     java.lang.String equalityConditionValue)
```

:   Gets sql code for a select on one column of a table, with a where x like
    y condition specific for that column

    :   **Parameters:**: `table` - the table to select from: `column` - the column to select from: `similarityConditionValue` - the x value for the: where column like '%x%' condition. **Returns:**: sql code for a select on one column of a table, with a where-like condition specific for that column

---


### getSelectWhereSql

```
public static java.lang.String getSelectWhereSql(java.lang.String table,
                                                 java.lang.String column,
                                                 java.lang.String whereCondition)
```

:   gets sql code for a select on one column of a table, with a where
    condition

    :   **Parameters:**: `table` - the table to select from: `column` - the column to select from **Returns:**: the sql for a simple one column unique and ordered query

---


### getSimpleSelectSql

```
public static java.lang.String getSimpleSelectSql(java.lang.String table,
                                                  java.lang.String column)
```

:   gets sql code for a select on one column of a table

    :   **Parameters:**: `table` - the table to select from: `column` - the column to select from **Returns:**: the sql for a simple one column unique and ordered query

---


### getSqlForSelectLike

```
public static java.lang.String getSqlForSelectLike(java.lang.String table,
                                                   java.lang.String column,
                                                   java.lang.String likeColumn,
                                                   java.lang.String likeCondition)
```

:   Sql code for a select "LIKE"

    :   **Parameters:**: `table` - the table to select from: `column` - the column to select from: `likeColumn` - the x variable for the "where x like y" sql statement: `likeCondition` - the y variable for the "where x like y" sql statement **Returns:**: an array with the selected results

---


### getSqlSelectByIdByRegExp

```
public static java.lang.String getSqlSelectByIdByRegExp(java.lang.String table,
                                                        java.lang.String valueColumn,
                                                        java.lang.String idColumn,
                                                        java.lang.String id,
                                                        java.lang.String regExp)
```

:   Generates the sql command for a select of a value corresponding to a
    certain id AND matching a certain regex string

    :   **Parameters:**: `table` - the table to select from: `valueColumn` - the column to select data from: `idColumn` - the column that contains the id to select by: `id` - the single id to select by: `regExp` - a regular expression pattern (check your database syntax for details) **Returns:**: an array with the selected results

---


### getSqlSelectByRegExp

```
public static java.lang.String getSqlSelectByRegExp(java.lang.String table,
                                                    java.lang.String column,
                                                    java.lang.String regExp)
```

:   Generates the sql command for a select matching a certain regex string

    :   **Parameters:**: `table` - the table to select from: `column` - the column to select data from: `regExp` - a regular expression pattern (check your database syntax for details) **Returns:**: the string with the sql statement text

---


### getSqlToDropTable

```
public static java.lang.String getSqlToDropTable(java.lang.String tableName)
```

:   Get sql to drop table

    :   **Returns:**: the drop table sql command

---


### surroundWith

```
public static java.lang.String surroundWith(java.lang.String text,
                                            java.lang.String surrounding)
```

:   Adds a surrounding string (usually an apex or similar) as prefix and
    suffix of the given text.

    :   **Parameters:**: `text` - string to be surrounded: `surrounding` - a string to use as prefix and suffix (for example, an apex) **Returns:**: the text surrounded by the


---


|  |  |  |
| --- | --- | --- |
| **Overview** \| **Package** \| **Class** \| **Use** \| **Tree** \| **Deprecated** \| **Index** \| **Help** | |  |
| **PREV CLASS**   **NEXT CLASS** | **FRAMES**    **NO FRAMES**     **All Classes** |  |
| SUMMARY: NESTED \| FIELD \| CONSTR \| METHOD | DETAIL: FIELD \| CONSTR \| METHOD |  |


---
